# Microbiota *in vitro* modulated with polyphenols shows decreased colonization resistance against *Clostridioides difficile* but can neutralize cytotoxicity

**DOI:** 10.1038/s41598-020-65253-0

**Published:** 2020-05-20

**Authors:** Aleksander Mahnic, Jennifer M. Auchtung, Nataša Poklar Ulrih, Robert A. Britton, Maja Rupnik

**Affiliations:** 1 National Laboratory for Health, Environment and Food, Prvomajska 1, 2000 Maribor, Slovenia; 2 Baylor College of Medicine, 1 Baylor Plaza, Houston, TX 77030 USA; 3 Present Address: University of Nebraska-Lincoln, Lincoln, NE 68588 USA; 4 University of Ljubljana, Biotechnical Faculty, Jamnikarjeva 101, 1000 Ljubljana, Slovenia; 5 University of Maribor, Faculty of Medicine, Taborska 8, 2000 Maribor, Slovenia

**Keywords:** Microbiome, Clostridium difficile

## Abstract

While the knowledge on gut microbiota - *C. difficile* interactions has improved over the years, the understanding of the underlying mechanisms providing colonization resistance as well as preventative measures against the infection remain incomplete. In this study the antibiotic clindamycin and polyphenol extracts from pomegranate and blueberries were used individually and in combination to modulate fecal microbial communities in minibioreactor arrays (MBRA). Modulated communities were inoculated with *C. difficile* (ribotype 027). Subsequent 7-day periodical monitoring included evaluation of *C. difficile* growth and activity of toxins TcdA and TcdB as well as analysis of MBRA bacterial community structure (V3V4 16 S metagenomics). Polyphenols affected multiple commensal bacterial groups and showed different synergistic and antagonistic effects in combination with clindamycin. Exposure to either clindamycin or polyphenols led to the loss of colonization resistance against *C. difficile*. The successful growth of *C. difficile* was most significantly correlated with the decrease in *Collinsella* and *Lachnospiraceae*. Additionally, we demonstrated that *Clostridium sporogenes* decreased the activity of both *C. difficile* toxins TcdA and TcdB. The feature was shown to be common among distinct *C. sporogenes* strains and could potentially be applicable as a non-antibiotic agent for the alleviation of *C. difficile* infection.

## Introduction

*Clostridioides difficile* infection (CDI) is a toxin mediated inflammation leading to diarrhoea and colitis, most commonly occurring in the hospital environment. A disturbed balance in the gut microbiota, usually resulting from antibiotic treatment, is needed for a successful colonization of the gastrointestinal tract (GIT) with *C. difficile* and the development of the infection^1^.

The gut microbiota provides colonization resistance against *C. difficile* via different mechanisms. Inhibition of growth or spore germination can be achieved through biotransformation of primary bile acids^2–4^, as well as pH reduction through organic acid production^5,6^. Other modes of action include the alteration of adhesion of *C. difficile* to enterocytes^7^, co-aggregation of probiotic strain (e.g. *Lactobacillus reuteri*) with pathogen^8^ or simply by competing for the adhesion sites. Selected fungal and bacterial strains were shown to decrease the activity of *C. difficile* toxins TcdA and TcdB. In 1996, a serine protease was characterized from *Saccharomyces boulardii* that inhibits toxin TcdA^9^, while more recently a protease with a similar effect was described in *Bacillus clausii*^10^. Disruption of *C. difficile* quorum sensing, a mechanism crucial for the regulation of toxin synthesis, can also lead to a decrease in toxin activity^11,12^.

Polyphenols are secondary plant metabolites found in abundance in food including different fruits, vegetables, seeds and herbs as well as in drinks such as coffee, tea and wine^13^. They are currently extensively researched because of their anti-oxidant and anti-inflammatory properties^14^, but also exhibit beneficial effects on commensal bacterial groups while inhibiting the growth of potential pathogens, including *C. difficile*^15,16^. Both blueberry and pomegranate extracts used in this study were previously reported to promote the growth of gut commensal bacteria and inhibit potential pathogens^17–19^ or modulate gut community towards beneficial outcomes^20–22^.

The aim of this study was to test if pre-exposure to polyphenols under *in vitro* conditions could affect microbial communities leading to the alleviation of the negative effects of antibiotics and subsequent improvement of colonization resistance against *C. difficile*. The *in vitro* system (MBRA) used in this study was previously shown to be suitable for studies concerning *C. difficile* – microbiota interactions^23^.

## Results

### **Different polyphenols and clindamycin modulate the bacterial community in distinct ways**

In the set of 24 mini-bioreactors (MBRAs) seeded with a pooled fecal sample from two healthy subjects, 3 were treated with clindamycin alone, 3 with a different polyphenol extract (PE, blueberry or pomegranate) and 3 with each polyphenol in combination with clindamycin (see Materials and Methods, Fig. 5). Pomegranate PE was used as a modulating factor at two concentrations, 100 mg/L and 400 mg/L. Dose dependant changes are shown in the Supplementary Fig. S1, while only the results obtained with modulation at the concentration of 400 mg/L are presented in the main article. Three non-treated bioreactors were used as a control.

**Figure 1:**
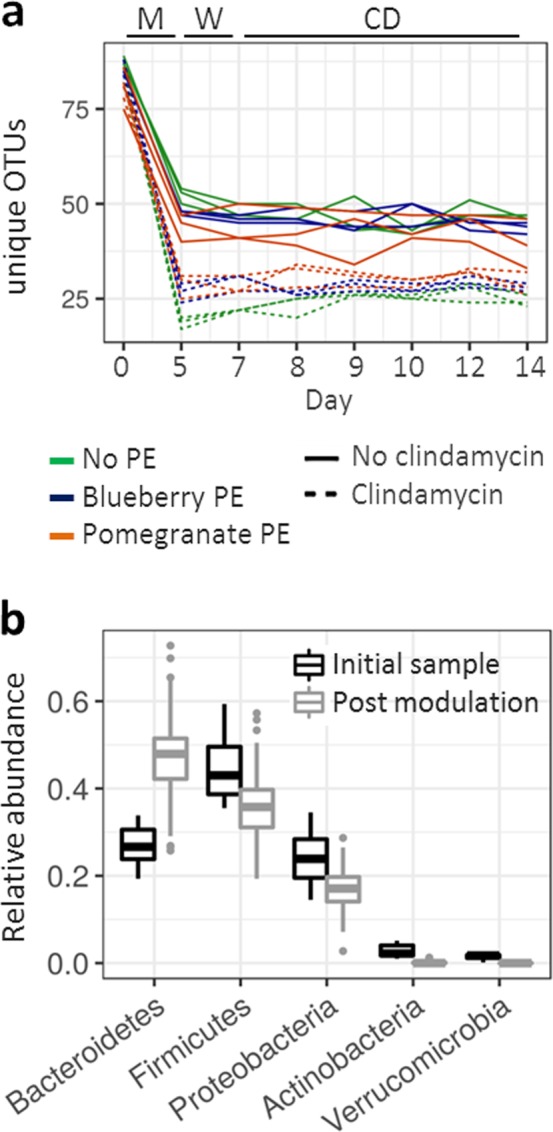
Bioreactors supported the growth of stable communities. **a** Number of unique OTUs observed throughout the experiment duration. Color indicates treatment with polyphenol extract (PE), dotted line indicates treatments where clindamycin was used as modulating factor. Letters on the top indicate experimental phase: M – modulation period, W – wash-out period and CD – post *C. difficile* inoculation period. **b** Box presentation of the relative abundance of bacterial phyla shown for the samples before the flow initiation (before flow, black) and for all remaining time points (after flow initiation, grey).

After a five-day microbiota incubation (Fig. 1a, day 5), we observed an expected decrease in bacterial richness in all treatments compared to the samples before the flow initiation (day 0; Fig. 1a). In subsequent time points the number of OTUs remained stable until experiment termination (14 days; Fig. 1a). Compared to the samples taken before the initiation of the flow, we observed a post-modulation increase in the relative abundance of different representatives of *Bacteroidetes* and the reduction of the remaining four major phyla found in the gut (Fig. 1b). Some key gut microbiota commensals could not be detected in the system after the modulation phase, including *Bifidobacterium* (OTU46 and OTU79), *Akkermansia* (OTU34) and multiple representatives of *Clostridiales*, most prominently *Faecalibacterium* (OTU39). These were present in the initial samples before the initiation of the flow at the average relative abundance of 0.8% (OTU46), 0.2% (Out79), 1.5% (OTU34) and 1.2% (OTU39). A prior study using the MBRA system similarly reported the decrease or disappearance of certain *Clostridiales* members including *Faecalibacterium* during the growth under similar continuous culture conditions, however in this case *Akkermansia* successfully grew in the system^24^.

**Figure 2:**
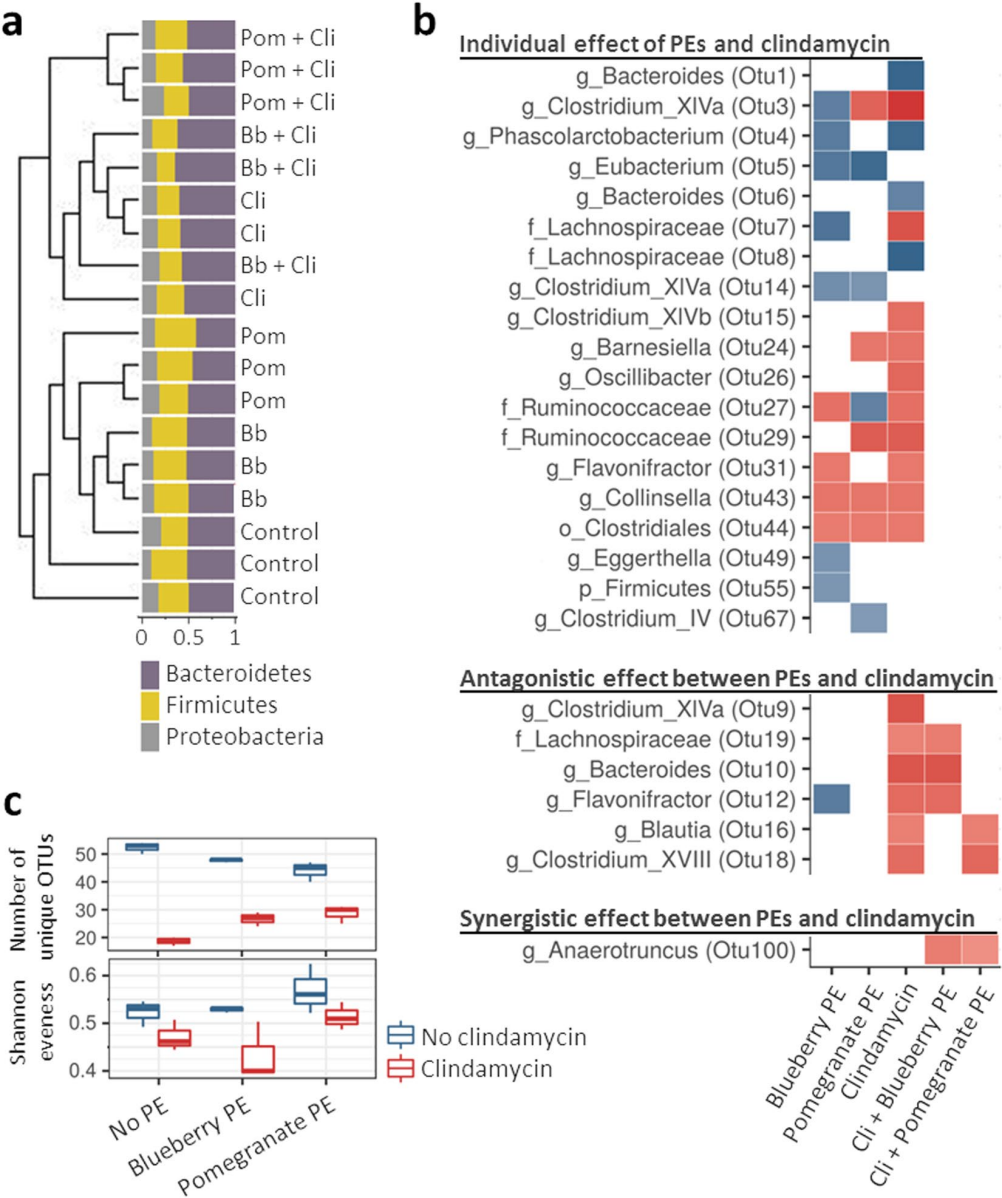
Polyphenols and clindamycin modulated microbiota in distinct ways. **a** Dendrogram presentation of Bray-Curtis dissimilarities with the bar-plot presentation of the relative abundance of 3 dominant bacterial phyla. Sample names are colored according to polyphenol extract (PE), that was used during the modulation phase. Green indicates control treatment, blue exposure to blueberry PE and orange exposure to pomegranate PE. Abbreviation “Cli” indicates exposure to clindamycin. **b** LEfSE analysis showing the comparison between communities exposed to either PE, clindamycin or combination of both compared to the control treatment. LDA values are presented as heat-plot with red indicating a decrease of specific OTU while blue an increase. Letter before OTU taxonomy indicates taxonomic level: g - genus, f - family, o - order, p - phylum. **c** Box presentation of the number of unique OTUs and Shannon evenness according to the treatment.

Microbial composition was most affected by modulation with clindamycin (AMOVA, p < 0.001). Clindamycin exposed microbiota profiles grouped together, while communities treated with PE only grouped closer to the untreated controls (Bray-Curtis dissimilarity dendrogram, Fig. 2a). However, only minor clindamycin-associated changes were observed at the phylum level (Fig. 2a), most prominently the increase in *Bacteroidetes*/*Firmicutes* ratio (p < 0.001). The most significant differentially represented OTUs are shown in Fig. 2b, while the complete LEfSe analysis is presented in Supplementary Fig. S1.

The highest number of unique OTUs was detected in the untreated control. Clindamycin as well as PEs decreased bacterial richness when applied alone (number of unique OTUs, p < 0.001; Fig. 2c). Interestingly, in both combinations of clindamycin and PE (clindamycin + blueberry PE and clindamycin + pomegranate PE) the number of detected OTUs decreased less compared to the bioreactors treated with clindamycin only (p < 0.001; Fig. 2c). Treatment with the pomegranate PE led to an increase in community evenness both alone and in combination with clindamycin (Shannon evenness, p < 0.001; Fig. 2c). Treatment with blueberry PE on the other hand further decreased community evenness when used in combination with clindamycin (Shannon evenness, p < 0.001; Fig. 2c).

Blueberry and pomegranate PEs modulated the bacterial community in distinct ways (AMOVA, p < 0.001). Several changes in microbial structure were however common to both PEs when compared to the control treatment. These included the increase in *Eubacterium* (OTU5) and *Clostridium* XIVa (OTU14) and decrease in *Collinsella* (OTU43) and *Clostridiales* (OTU44). Other differentially represented OTUs were PE specific. Most prominently, modulation with blueberry PE led to an increase in *Clostridium* XIVa (OTU3), *Lachnospiraceae* (OTU7), *Flavonifractor* (OTU12), *Eggerthella* (OTU49) and unclassified *Firmicutes* (OTU55). Modulation with pomegranate PE led to an increase in *Ruminococcaceae* (OTU27) and *Clostridium IV* (OTU67) (Fig. 1b). The complete LEfSe analysis is shown in Supplementary Fig. S1.

The treatment of microbiota with PEs and clindamycin in combination resulted in specific patterns indicating antagonistic and synergistic effects between the two. Most noteworthy we found bacterial groups which were decreased after the exposure to clindamycin alone but were unaffected when PEs were used in combination with clindamycin. Among these, blueberry PE minimized the adverse effect of clindamycin on *Blautia* (OTU16) and *Clostridium XVIII* (OTU18). Pomegranate PE minimized the adverse effect of clindamycin on *Bacteroides* (OTU10), *Flavonifractor* (OTU12) and *Lachnospiraceae* (OTU19). Both blueberry and pomegranate PEs alleviated the adverse effect of clindamycin on *Clostridium XIVa* (OTU9). On the other hand, we also observed a synergistic effect of PEs and clindamycin. A combination of clindamycin and either PE resulted in a decrease in *Anaerotruncus* (OTU100) which was unaffected when either PE or clindamycin were used individually (Fig. 2b). Pomegranate effects at the lower PE concentration (100 mg/L) were in concordance with results observed at PE concentration of 400 mg/L (Supplementary Fig. S1).

### **Colonization resistance to*C. difficile*decreased after polyphenol exposure**

After the modulation period we performed a 2 day wash out period in order to allow the communities to stabilize and to wash the modulation factors (PEs and clindamycin) out of the system. From this point on the bioreactors were supplied with medium without modulation factors. Subsequently, on day 7 we inoculated all bioreactors with *C. difficile* vegetative cells at the final concentration of approximately 10^5^ CFU/mL. The growth and cytotoxicity of *C. difficile* were periodically measured for the next 7 days.

**Figure 3:**
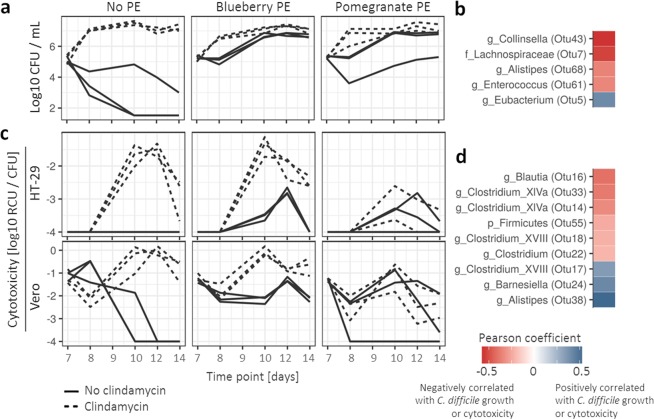
*C. difficile* growth- and cytotoxicity-associated bacterial community characteristics. Timeline of *C. difficile* growth (log10 CFU/mL) **(a)** and cytotoxicity (log10 Relative Cytotoxicity Units (RCU)/*C. difficile* CFU) shown for cell lines HT-29 and Vero, separately **(c)**. Dotted line indicates exposure to clindamycin during the modulation phase. **b,d** Pearson analysis of OTU correlation with *C. difficile* growth **(b)** and cytotoxicity **(d)**. Red color indicates negative correlation with either *C. difficile* growth or cytotoxicity while blue indicates a positive correlation.

As expected, control (untreated) microbial communities exhibited colonization resistance against *C. difficile* while in communities that were treated with clindamycin during the modulation period *C. difficile* grew successfully (Fig. 3a). We hypothesised that PEs will modulate microbiota towards an increased resilience against clindamycin induced changes resulting in colonization resistance in bioreactors exposed to the combination of PE and clindamycin. Contrary to our expectations, *C. difficile* grew successfully in all treatments exposed to PEs (alone and in combination with clindamycin) (Fig. 3a).

Several community characteristics correlated significantly with *C. difficile* growth (Fig. 3b). The number of unique observed OTUs (community richness) was negatively correlated with *C. difficile* concentration (Pearson’s r = −0.49, p-value <0.001). The two OTUs most significantly negatively correlated with *C. difficile* growth were *Collinsella* (OTU43; Pearson’s r = −0.56, adjusted p-value <0.001) and *Lachnospiraceae* (OTU7; Pearson’s r = −0.53, adjusted p-value <0.001) (Fig. 3b). The OTU most significantly positively correlated with *C. difficile* growth was *Eubacterium* (OTU5; Pearson’s r = 0.41, adjusted p-value = 0.035) (Fig. 3b). No correlation was observed with overall bacterial diversity (Shannon index). Detailed presentation of correlation patterns is available in Supplementary Fig. S2. Additionally we report that neither PE used in this study exhibited any direct effect on *C. difficile* growth or cytotoxicity at concentrations lower than 2000 mg/L (Supplementary Fig. S3).

### **A subset of the PE modulated microbial communities reduced cytotoxicity but did not affect*C. difficile*growth**

Cytotoxicity of bioreactor supernatants was measured by performing tests on two cell lines, HT-29 (more sensitive to TcdA) and Vero (more sensitive to TcdB). Measurements from both cell lines were correlated although the values were significantly lower for the HT-29 cell line (Fig. 3c). Two days after *C. difficile* inoculation we additionally quantified TcdA and TcdB separately with an ELISA assay and the results are in congruence with the cytotoxicity assays (Supplementary Figure S4).

The activity of toxins correlated with *C. difficile* growth (Pearson’s r = 0.33 and 0.27, p-value = 0.001 and 0.009 for HT-29 and Vero, respectively). The exception was the bioreactor in which the microbiota was exposed to pomegranate PE in combination with clindamycin (Fig. 3c). Here, the relative cytotoxicity units per *C. difficile* CFU (RCU/*C. difficile* CFU) were significantly lower in comparison to other treatments, which were exposed to clindamycin during the modulation phase (p < 0.001). This indicated good *C. difficile* growth but a decrease in cytotoxicity. Using the Pearson correlation test we identified OTUs which were correlated with the reduction in cytotoxicity (Fig. 3d). We found that among the OTUs listed in Fig. 3d, an increase in either *Clostridium* (OTU22) or *Blautia* (OTU16) corresponded to bioreactor specific reduction in cytotoxicity to the largest extent (Supplementary Figure S5).

### ***C. sporogenes*decreases the activity of toxins TcdA and TcdB but does not impact*C. difficile*growth**

From the bioreactor content, we successfully isolated four bacterial strains which corresponded to the OTUs correlated with the decrease in cytotoxicity. Using 16 S rRNA gene sequencing (Sanger method) we identified these to be two distinct *Clostridium sporogenes* strains (MBRA strain 1 and 2), *Clostridium oroticum* and *Blautia sp*. strain, corresponding to OTUs *Clostridium* (OTU22), a *Clostridium* XIVa (OTU33) and a *Blautia* (OTU16), respectively.

**Figure 4:**
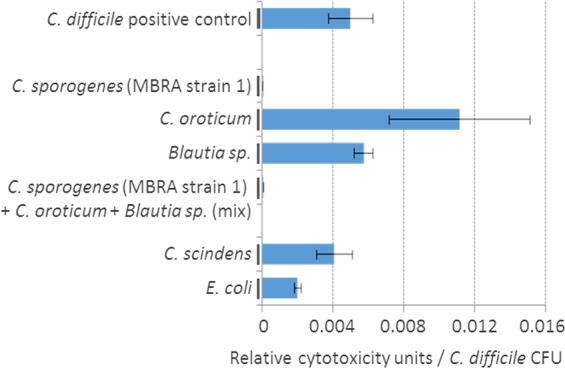
*C. difficile* cytotoxicity per CFU after co-culture with different bacterial strains. *C. sporogenes, C. oroticum* and *Blautia sp*. strains were isolated from MBRA content while *Clostridium scindens* and *E. coli* were used as controls. Strains were tested in co-culture with *C. difficile*. Graph presents average measurements of relative cytotoxicity units (Vero cells) per *C. difficile* CFU ± SD (n = 3).

Isolated strains were co-cultured with *C. difficile* to test their impact on *C. difficile* growth and cytotoxicity. The *C. sporogenes* strain (MBRA strain 1) was able to decrease the activity of toxins per *C. difficile* CFU (p < 0.001) while no impact on *C. difficile* growth was observed. No such effect was observed with *Blautia sp*. and *C. oroticum* (Fig. 4). We additionally tested 7 distinct environmental *C. sporogenes* strains and observed the inhibitory effect towards both *C. difficile* toxins TcdA and TcdB in all instances (Supplementary Figure S6).

## Discussion

In the present study we have shown that an *in vitro* modulation of human fecal microbial communities in MBRAs can lead to different *C. difficile* colonization-associated outcomes in a replicable manner. Contrary to our expectations, modulation with PEs decreased colonization resistance against *C. difficile*. Growth of *C. difficile* and the activity of toxins TcdA and TcdB were correlated with specific bacterial groups in a complex community. Further, we have demonstrated a direct inhibitory effect of *C. sporogenes* on both *C. difficile* toxins TcdA and TcdB.

Blueberry and pomegranate PE modulated microbiota in a distinct manner, which was only partially in concordance with previous published data^18,21^. Discrepancies can result from different polyphenol extract composition and concentration as well as different microbial community and bioreactor characteristics, making these studies difficult to compare. Contrary to our expectations both polyphenols modulated the microbial community towards the loss of colonization resistance against *C. difficile*. The direct inhibitory effects of polyphenols on *C. difficile* growth were previously reported^16^. However, no previous studies concerning *C. difficile* colonization in polyphenol modulated microbial communities (*in vivo* or *in vitro*) could be found. Unrelated to *C. difficile*, studies predominantly reported beneficial effects of polyphenols on commensal microbiota^13^. However, our findings suggest that polyphenols can have an adverse effect on some commensal bacterial groups, most importantly different representatives from *Clostridiales*.

An important aspect is the concentration of supplemented polyphenols and the mode of delivery. Concentrations used in our study were calculated as an approximation to a daily consumption of one tea spoon of polyphenol extract in relation to an average lumen of an adult gastrointestinal tract. Similar concentrations were also used by others in the past^25–27^. Also we supplied polyphenol extracts at a low dose continuously in the media as oppose to the ingestion of the daily dose at once. Overall, it is difficult to simulate daily consumed doses of polyphenol extract under laboratory conditions. Further concentration and application dependent effects of polyphenol extract as well as *in vivo* studies are therefore required to assure safe implementation of polyphenols in consumable products.

Both polyphenols showed synergistic and antagonistic effects with clindamycin towards different bacterial groups, mainly representatives from *Clostridiales*. Antagonistic effects are rarely reported^28–30^. Synergistic effects are, on the other hand, more common and interesting because of the potential to decrease antibiotic doses during infection treatment^31–33^. These effects are commonly investigated on common pathogens; however, they should also be investigated on commensal bacterial groups. Adverse synergistic effects affecting commensal clostridia are interesting for future research because of their potential to stimulate the development of dysbiosis in susceptible subjects.

The development of microbial communities was bioreactor-specific, diverging also among replicates of the same treatment, as previously reported^24^. In the referenced study the authors discussed development and stability of communities in MBRA as well as possible reasons for differences among replicates into great detail. Nevertheless, *C. difficile* colonization- and cytotoxicity-associated outcomes were in our case treatment specific and bacterial community associations were highly significant.

Representatives of *Lachnospiraceae* and *Collinsella* were most prominently associated with the inhibition of *C. difficile* growth, both in concordance with previously reported data. Members of *Lachnospiraceae* were highlighted as protective against development of CDI in patients after allogeneic hematopoietic stem cell transplantation^34^ and were able to alleviate CDI-associated symptoms in germfree mice^35^. *Collinsella* was previously reported to be decreased in CDI patients^36^. Also, both *Lachnospiraceae* and *Collinsella* are known to participate in the biotransformation of bile acids^37–39^. Despite this, the exact mechanism responsible for the observed associations with *C. difficile* growth in our *in vitro* system could not be clarified with the available data.

Previously reported bacteria and fungi mediated suppression of *C. difficile* toxins^9,10,40,41^ does not include *C. sporogenes*. Suppression of toxin activity mediated by a heat sensitive secondary metabolite has been observed for *Lactobacillus delbrueckii*^40^*, L. lactis*^41^ and *Bacillus clausii*^10^. A serine protease producing *Saccharomyces boulardii*^9^ was shown (as a sole probiotic strain as well as in combination with bacterial probiotic strains) to be effective at the prevention of primary CDI^42^. However, probiotics with fungi are not recommended for pregnant women and immune-compromised patients because of an elevated risk of fungemia^43^. Commensal bacterial strains such as *C. sporogenes* could in such cases present a safer alternative. We have not further examined the molecular basis of the observed activity.

In conclusion, exposure of microbiota to blueberry and pomegranate PEs led to a decrease of colonization resistance against *C. difficile*. Future *in vivo* studies are required; however, our results indicate that polyphenols are strong microbiota modulators that also exhibit synergistic and antagonistic effects with clindamycin and should therefore be further investigated for a safer use. In spite of this decrease in colonization resistance, pomegranate PEs in some instances inhibited *C. difficile* toxin activity, likely by promoting expansion of *C. sporogenes. C. sporogenes* was further shown to neutralize the cytotoxicity of the main *C. difficile* toxins TcdA and TcdB.

## Materials and methods

### **Preparation of polyphenolic extracts**

Ripe blueberries (*Vaccinium myrtillus* L.) were sampled in Slovenia and stored for 7 days at −20 °C prior to the beginning of the extraction process. The method has been already described^44^ and is briefly presented here. Frozen samples (50 g) were homogenized in 150 mL of ice-cold deoxygenated methanol, previously flushed for a few minutes by nitrogen. Homogenate was extracted for 3 h by shaking (Shaker EV403, Tehtnica, Zelezniki, Slovenia) in the dark at room temperature. The extract was centrifuged and the supernatant was stored at − 20 °C. The sediment was extracted again in 100 mL of deoxygenated methanol for 2 h in the dark at room temperature and the suspension was centrifuged. To completely remove the methanol after polyphenol extraction, the sample was first dried in a Speed-Vac, then frozen at −80 °C and lyophilized. With this procedure we completely remove the organic solvents. Finally, both supernatants were pooled, flushed with nitrogen for a few minutes and then stored at − 20 °C until analyzed^44^.

Pomegranate (*Punica granatum* L.) fruits were harvested in the normal ripening period in Istria (Marasi). The pomegranate seeds or arils were separated from the peel and mesocarp. Different parts of pomegranate (mesocarp, squeezed juice from arils and peel) were lyophilized and crushed to powder^45^. Here we used the ethanol extracts from dry peels prepared by extracting lyophilized powdered peels in 70% ethanol for four hours. The extracts were centrifuged and dried using rotary evaporation and lyophilization. Extracts were kept at −20 °C until needed. For experiments all extracts were dissolved in distilled sterile water to avoid the additional effect of alcohol on microbiota

### **Minibioreactor arrays (MBRAs) experiment setup**

The MBRA setup design and medium preparation used in this study is described in great detail in Auchtung *et al*.^46^. Importantly, MBRAs allow a simultaneous running of 24 independent continuous flow bioreactors. Bioreactors have an internal volume of 25 mL and working volume of 15 mL. The MBRAs were set in an anaerobic chamber under anaerobic atmosphere (5% CO2–5% H2–90% N2) at a constant temperature of 37 °C. The media was continuously replenished, and waste was removed at a flow rate of 1.875 mL/h. MBRA system is stationed on the magnetic stir plate allowing for a constant stirring of the bioreactor slurry during operation.

Bioreactors were inoculated with a pooled stool sample from two subjects. The two fecal samples were randomly selected from a bank of samples of anonymous donors between the ages of 18–65 who self-identified as healthy and had not consumed antibiotics in the previous 6 months; the pool of anonymous donors included samples from male and female donors. Stool sample collection and preparation before inoculation is described in Auchtung *et al*.^24^. Stool sample collection was reviewed and approved by the Institutional Review Board from Michigan State University and all the study procedures were carried out in accordance with relevant guidelines. All individuals donating samples provided informed consent prior to donation.

**Figure 5:**
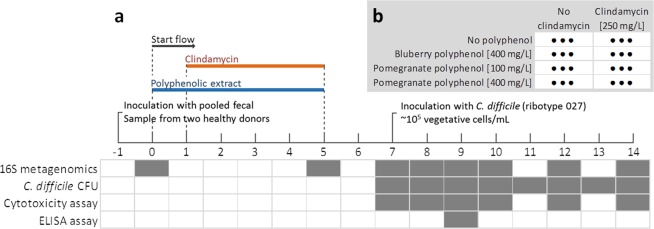
Schematic presentation of the experiment design. **a** Time progression of the experiment with the events noted on the timeline. Table below timeline shows at which time points were different tests performed. **b** The setup of 24 bioreactors (8 unique treatments each done in triplicate) according to the modulation factors used.

After inoculation the communities were left to stabilize in MBRAs for 24 h without flow (Fig. 5a). Prior publication using the same system determined that the period of 24 h was sufficient for stabilization based on the changes in microbial diversity^24^. After the flow initiation, bioreactors were supplied with different combinations of modulating factors added into the BDM medium^46^ resulting in 8 unique treatments, each done in a triplicate (Fig. 5b). Reactor medium was buffered with phosphate buffer and 5% CO_2_/0.2% bicarbonate buffering system. Previous experiments have demonstrated that this buffering system is sufficient to buffer from pH changes due to metabolism of 10X excess of complex carbohydrates compared to what was used in the medium. Antibiotic clindamycin and two polyphenolic extracts (PE; blueberry and pomegranate) were used as modulating factors. Physico-chemical transformation of media and modulating factors during the supplementation of bioreactors was not investigated. Clindamycin was used at a final concentration of 250 mg/L, blueberry polyphenolic extract was used at a final concentration of 400 mg/L and pomegranate polyphenolic extract was used at two concentrations, 100 mg/L and 400 mg/L. Solution of polyphenolic extracts was prepared in dH_2_O. The modulation with PEs was initiated simultaneously with the flow for the next 5 days, while the modulation with clindamycin was initiated 1 day later for the next 4 days (Fig. 5a).

After the modulation period, MBRAs were supplied with BDM medium for 2 days (wash out period) in order to stabilize communities and wash out the modulating factors from bioreactor content. Subsequently we inoculated all bioreactors with the *C. difficile* culture (CD2015; ribotype 027) at the final concentration of approximately 10^5^ vegetative cells/mL. For the next 7 days each bioreactor was periodically sampled. We tested the *C. difficile* growth, sporulation and cytotoxicity as well as bacterial community composition (Fig. 5a).

We did not measure the basal concentrations of polyphenols in the donor’s feces or measure the polyphenols-derived metabolites produced in the bioreactors.

### **Measurement of*C. difficile*cell concentration**

Total number of colony forming units (CFU) was determined by plating serial dilutions of bioreactor content onto the *C. difficile* selective media TCCFA with 50 μg/ml rifampicin and 20 μg/ml erythromycin.

### **Cytotoxicity test for semi-quantitative measurement of the activity of*C. difficile*toxins TcdA and TcdB**

A cytotoxicity test was performed on HT-29 cells (more sensitive to TcdA) and Vero cells (more sensitive to TcdB). Frozen culture (−80 °C) of either cell line HT-29 or Vero was thawed in a water bath (37 °C for 5 min). The content (1 mL) was combined with 5 mL of fresh medium (DMEM (Gibco) + 10% fetal bovine serum (FBS) at 37 °C) in the cell culture flask (25 cm^2^). Cell culture was incubated at 5% CO_2_ and 37 °C. After the formation of a monolayer, cells were trypsinized and transferred to 75 cm^2^ flasks with 9 mL of DMEM (Gibco) + 10% FBS and incubated at 5% CO_2_ and 37 °C. Before preparing mature cells for the assay, cells were centrifuged (5000 rpm for 5 min), counted to determine concentration and diluted with fresh DMEM (Gibco) + 10% FBS to the final concentration of 5 × 10^4^ cells /mL. The suspension was then aliquoted in the 96-well plate (100 μL per well) and incubated for 24 h at 5% CO_2_ and 37 °C.

Supernatants for the assay were prepared by first centrifuging the bioreactor content (20000 rpm for 20 min) and a subsequent filtration of the supernatant (0.2 μM filter, PES membrane). The five-fold serial dilutions of supernatant were added to the formed cell monolayer and incubated 24 h at 5% CO_2_ and 37 °C.

The scoring of the cytotoxic effect was determined as follows: 1) no cell rounding effect (0 points), 2) from approximately 30% to 70% of cells were rounded (0.5 points) and 3) more than 70% of cells were rounded (1 point). Each score was subsequently used as an exponent to the base 10 to obtain relative cytotoxicity units (RCUs).

### **Detection of*C. difficile*toxins TcdA and TcdB with commercial test**

Each toxin separately was additionally quantified with the TGC-E002–1 kit (tgcBIOMICS, Germany) according to the recommended protocol. The concentration of each toxin was determined based on a standard dilution curve, obtained with purified toxins TcdA and TcdB provided in the kit.

### **16S rRNA gene sequencing of MBRA bacterial communities**

MBRA content samples (1 mL) were centrifuged at 14000 rpm for 1 min to obtain a pellet, which was stored at −80 °C until further use. Total DNA was extracted with QIAamp DNA Mini kit (QIAGEN) using a modified protocol. Pellets were re-suspended using 360 μL of buffer ATL and homogenized in MagnaLyser (Roche) at 7000 rpm for 70 s. Next, 40 μL of proteinase K was added followed by an incubation at 55 °C (1 h). In the next step 200 μL of buffer AL was added followed by an incubation at 70 °C (30 min). After the addition of 200 μL of 96–100% ethanol the content was transferred into column tubes and the subsequent steps followed the protocol provided in QIAamp DNA Mini kit. Extracted DNA was stored at −80 °C until further use.

Bacterial community structure was determined by sequencing the V3V4 variable region of the 16 S rRNA gene. Libraries were prepared according to the 16 S Metagenomic Sequencing Library Preparation (Illumina) protocol using the primer pair Bakt_341F (5′-CCTACGGGNGGCWGCAG-3′) – Bakt_805R (5′-GACTACHVGGGTATCTAATCC-3′) (approximately 460 bp fragment^47^). Sequencing was performed on the Illumina MiSeq platform (paired-end sequencing, 2 × 300 bp).

### **Sequence data analysis and statistics**

Quality filtering was performed using mothur (v.1.36.1)^48^ following the protocol recommended in Kozich *et al*.^49^. Silva reference base (Release 123) was used for the alignment. Chimeras were identified using mothur implemented UCHIME algorithm. Taxonomy was inferred using the RDP training set (v.12) provided by mothur implementing 0.80 bootstrap value.

We obtained a total of 7576427 reads (min: 14453, max: 81825, average per sample: 39460.56). We removed reads which were present at the overall abundance lower than 0.01% and rarefied the number of reads to 14000 reads per sample using random sampling implemented in mothur.

Downstream analysis was performed in mothur (calculation of alpha diversity indices (Shannon index, Shannon evenness index), beta diversity indices (Bray-Curtis and AMOVA testing) and population-level analysis (linear discriminant analysis effect size, LEfSe)^50^. Remaining statistics were performed in R environment (version 3.1.3)^51^ using packages ‘vegan’^52^. All graphical presentations were made in R environment with package ‘ggplot2′^53^ except Fig. 5, which was created in PowerPoint (Microsoft).

### **Testing the effect of different strains on*C. difficile*growth and cytotoxicity**

Three *C. difficile* strains were used in testing, all belonging to the ribotype 027. These included *C. difficile* strain used in the MBRA experiment (CD2015) and two strains from NLZOH collection (ZZV12-4777 and ZZV14-5907). *C. difficile* strains were tested in combination with four strains that were successfully isolated from bioreactor contents. These included two *C. sporogenes* strains, *C. oroticum* and *Blautia sp*. strains. Identification was performed with Sanger sequencing of the entire 16 S rRNA gene. Sanger sequencing was performed using the universal bacterial primer pair 27fevb (5′-GAGAGTTTGATCCTGGCTCAG-3′) – 1495revb (5′-CTACGGCTACCTTGTTACGA-3′) on the Applied Biosystems 3500 Series Genetic Analyzer (Thermo Fisher Scientific) according to the previously published protocol^54^. Sequences were subsequently compared to the MiSeq reads. With high certainty we were able to show that these strains correspond to *Clostridium* (OTU22), a *Clostridium* XIVa (OTU33) and a *Blautia* (OTU16), respectively. Growth curves for all strains tested were determined by absorbance measurement at OD 600 nm.

Co-culturing assay was performed in 6-well plates with 5 mL working volume. The overnight culture (24 h) of the *in vitro C. difficile* strain and the bacterial strain under investigation (*C. sporogenes, C. oroticum* and *Blautia sp*.) (50 μL) was added to 5 mL of anaerobic Wilkins-Chalgren Anaerobe Broth (WCAB). After 24 h incubation at 37 °C in anaerobic atmosphere we sampled 1 mL. Total CFU count was performed by plating serial dilutions on CHROMID *C. difficile* (BioMerieux). Cytotoxicity assay was performed as described above but only on cell line Vero.

Three *C. difficile* strains and nine *C. sporogenes* strains were additionally tested for the effect of cell-free *C. sporogenes* supernatants on *C. difficile* toxins TcdA and TcdB. Two *C. sporogenes* strains were isolated from bioreactor slurry (MBRA strain 1 and 2) while seven were obtained from NLZOH collection. *C. difficile* strains were cultured overnight (24 h). *C. sporogenes* strains were sampled after 48 h cultivation. Samples were filter sterilized (PES membrane, 0.2 μM, Sarstedt). Combinations of *C. difficile/C. sporogenes* supernatants (1:1 volume ratio) were incubated for 12 h at 37 °C in anaerobic conditions with shaking. TcdA and TcdB toxin activities were quantitatively measured with ELISA test (TGC-E002-1, tgcBIOMICS).

## Supplementary information

Supplementary information.

## Data Availability

The datasets supporting the conclusions of this article are available in the form of combined paired end reads (contigs) on the Metagenomics RAST (MG-RAST) database server (http://metagenomics.anl.gov/) under the project access number mgp90214 (https://www.mg-rast.org/mgmain.html?mgpage=project&project=mgp90214).
