# Supplementary material for: Microbiota in vitro modulated with polyphenols shows decreased colonization resistance against Clostridioides difficile but can neutralize cytotoxicity
Source: Sci Rep. 2020 May 20;10:8358. doi: 10.1038/s41598-020-65253-0 (PMC7239955; doi:10.1038/s41598-020-65253-0)
Supplement: Supplementary file 1 — Supplementary information. [file 41598_2020_65253_MOESM1_ESM.docx]

**Microbiota in vitro modulated with polyphenols** **shows decreased colonization resistance against *Clostridioides difficile* but can neutralize cytotoxicity**

Aleksander Mahnic^1^, Jennifer M. Auchtung^2,3^, Nataša Poklar Ulrih^4^, Robert A. Britton^2^, Maja Rupnik^1,5*^

^1^ National Laboratory for Health, Environment and Food, Prvomajska 1, 2000 Maribor, Slovenia

^2^ Baylor College of Medicine, 1 Baylor Plaza, Houston, TX 77030, USA

^3^ Present address: University of Nebraska-Lincoln, Lincoln, NE 68588, USA

^4^ University of Ljubljana, Biotechnical Faculty, Jamnikarjeva 101, 1000 Ljubljana, Slovenia

^5^ University of Maribor, Faculty of Medicine, Taborska 8, 2000 Maribor, Slovenia

*corresponding author

maja.rupnik@nlzoh.si


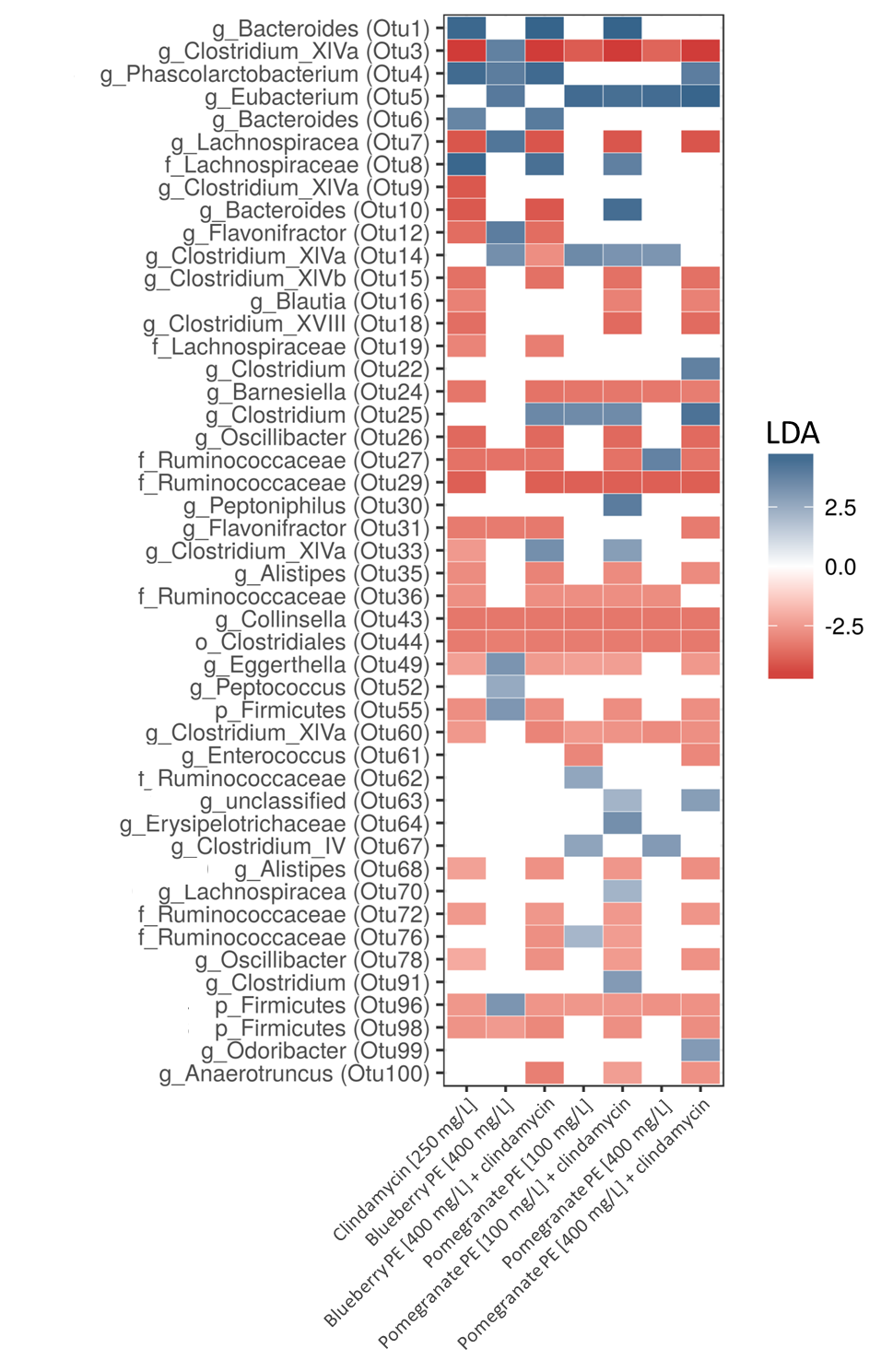


**Supplementary Figure S1. LEfSe analysis.** Colors present LDA values with red indicating decrease in comparison to control treatment and blue an increase.


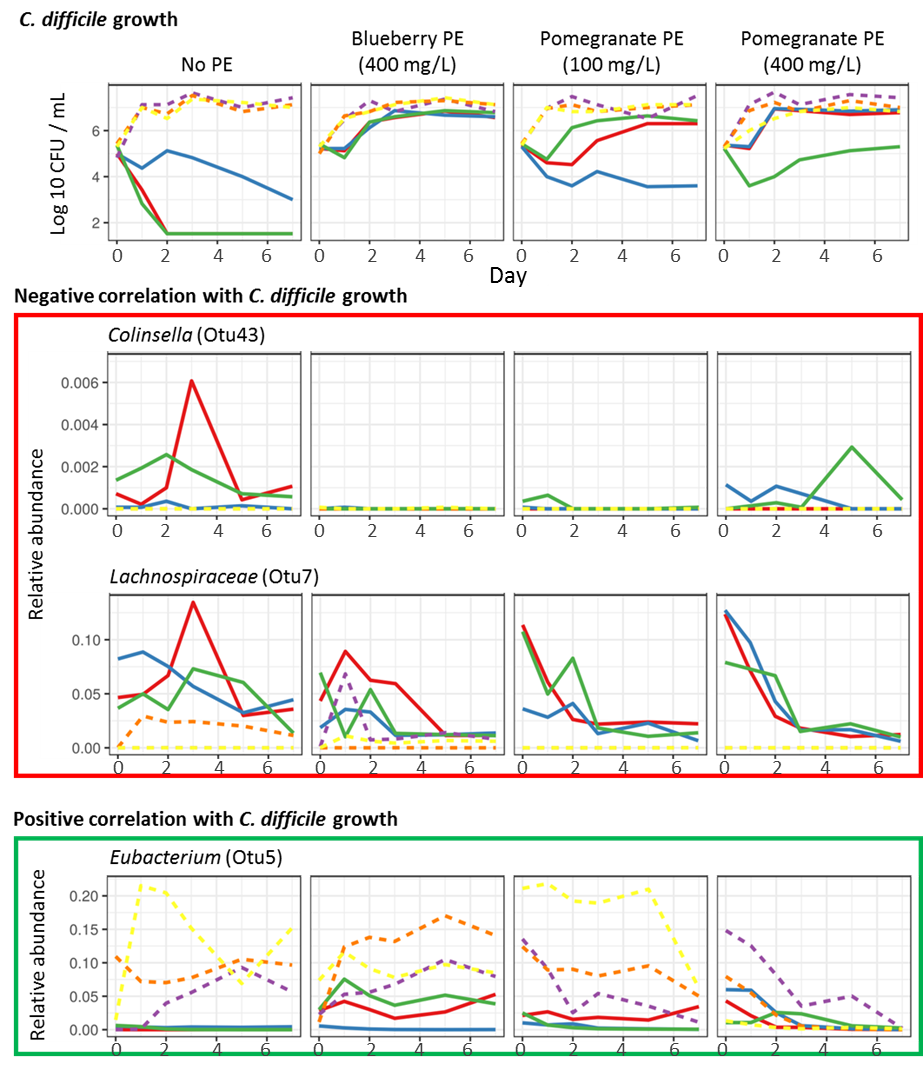


**Supplementary Figure S2. OTUs most significantly correlated with *C. difficile* growth.** Top-most graph presents concentration of *C. difficile* at different time-points (days) after inoculation of MBRA with *C. difficile* vegetative cells. Each line represents data from a replicate reactor; dashed lines indicate reactors treated with clindamycin and solid lines indicate reactors not treated with antibiotic. Concentration was obtained by plating on selective medium. The two OTUs highlighted in red bracket were significantly negatively correlated (Pearson correlation test) while OTU in green bracket was positively correlated with *C. difficile* growth. Graphs present their relative abundance obtained by 16S metagenomic analysis.

**
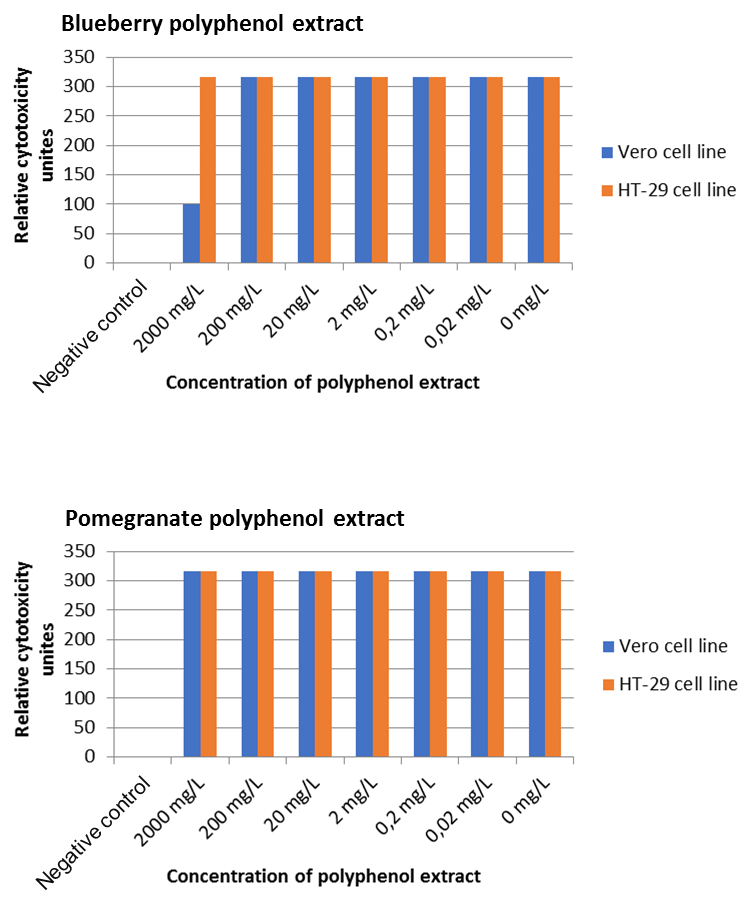
**

**Supplementary Figure S3. Effect of polyphenols on *C. difficile* cytotoxicity and Vero and HT-29 cells.** Graph shows relative cytotoxicity units obtained by cultivating *C. difficile* strain used in MBRA experiment in Wilkins-Chalgren anaerobe broth (WCAB) at different concentrations of bluberry (above) and pomegranate polyphenol extract (below). Negative control represents WCAB + polyphenol at 2000 mg/L with no *C. difficile* inoculated. Results show that no cytotoxicity effect was observed on either cell line in the presence of either polyphenol. Polyphenols on the other hand showed no effect on *C. difficile* cytotoxicity with the exception of the reduction of cytotoxicity measured with Vero cells in the sample treated with blueberry polyphenol at 2000 mg/L.


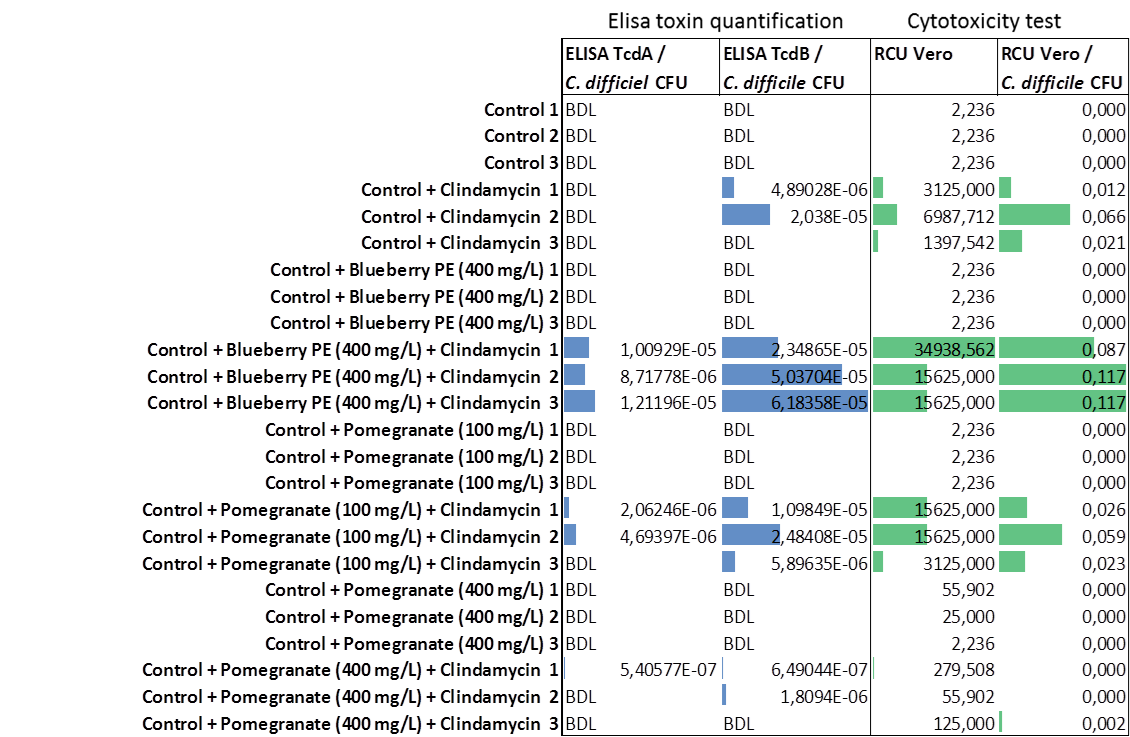


**Supplementary Figure S4. The amount of active toxin in MBRA supernatants at time point 9.** Comparison between results obtained with ELISA test and cytotoxicity test performed on Vero cell line shows that the trends are comparable between the two tests. This in combination with tests on filter steralized supernatants shows that the degraded toxins can not be recognized by antibodies used in commercial ELISA test.


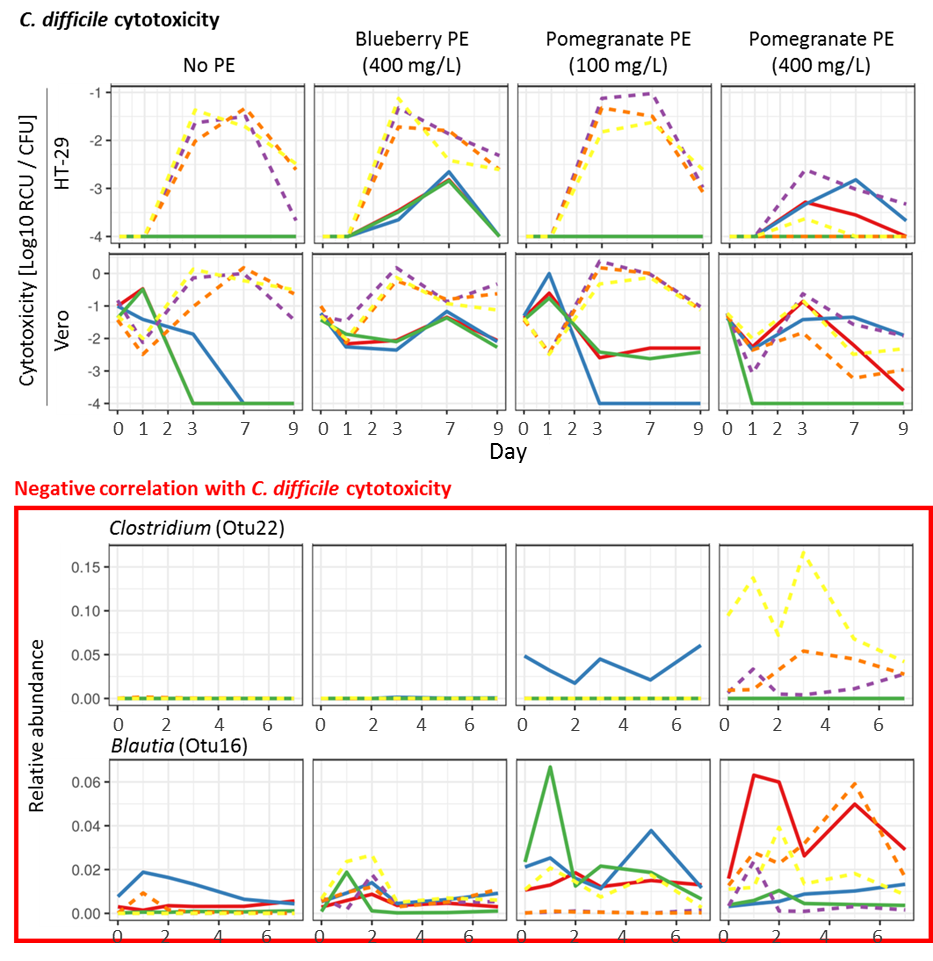


**Supplementary Figure S5. OTUs most significantly correlated with *C. difficile* cytotoxicity.** Top-most graph presents the cytotoxicity of *C. difficile* at different time-points (days) after inoculation of MBRA with *C. difficile* vegetative cells for both TcdA and TcdB. Each line represents data from a replicate reactor; dashed lines indicate reactors treated with clindamycin and solid lines indicate reactors not treated with antibiotic. The two OTUs highlighted in red bracket were significantly negatively correlated (Pearson correlation test) with *C. difficile* cytotoxicity. Graphs present their relative abundance obtained by 16S metagenomic analysis.


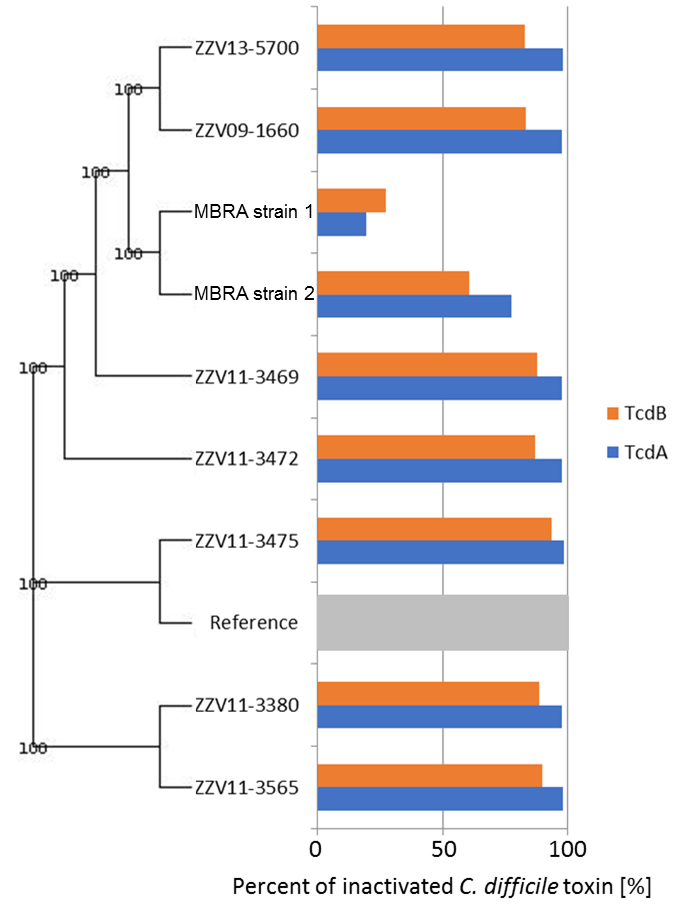


**Supplementary Figure S6. Percent of *C. difficile* toxin inactivated by *C. sporogenes* supernatant.** TcdA and TcdB were quantified with commercial test. Measurements for samples where *C. difficile* and *C. sporogenes* supernatants were incubated together (1:1 ration) were extracted from positive control (only *C. difficile* supernatants). Values are presented as percentage of TcdA (blue) and TcdB (orange) that got inactivated by *C. sporogenes* supernatants. Phylogenetic classification of *C. sporogenes* strains was obtained by whole genome sequencing. Reference is the representative *C. sporogenes* genome obtained from NCBI ([www.ncbi.nlm.nih.gov](http://www.ncbi.nlm.nih.gov/); 15.12.2019)
